# Supplementary material for: Acetylcholine modulates the temporal dynamics of human theta oscillations during memory
Source: Nat Commun. 2023 Aug 30;14:5283. doi: 10.1038/s41467-023-41025-y (PMC10469188; doi:10.1038/s41467-023-41025-y)
Supplement: Supplementary file 1 — Supplementary Information [file 41467_2023_41025_MOESM1_ESM.pdf]

Acetylcholine modulates the temporal dynamics of human theta oscillations during memory

Tamara Gedankien[1], Ryan Joseph Tan[2], Salman Ehtesham Qasim[3], Haley Moore[2], David McDonagh[4], Joshua Jacobs[1,5,6,*], and Bradley Lega[2,6,+]

[1]Department of Biomedical Engineering, Columbia University, New York, NY, 10027, USA
[2]Department of Neurological Surgery, University of Texas Southwestern, Dallas, TX, 75390, USA
[3]Department of Psychiatry, Icahn School of Medicine at Mount Sinai, New York, NY, 10029, USA
[4]Department of Anesthesiology, University of Texas Southwestern, Dallas, TX, 75390, USA
[5]Department of Neurological Surgery, Columbia University, New York, NY, 10032, USA
[6]these authors contributed equally to this work
[*]Correspondence: joshua.jacobs@columbia.edu
[+]Correspondence: bradley.lega@utsouthwestern.edu

SUPPLEMENTARY INFORMATION

Supplementary Discussion

Gene expression in the human hippocampus

The findings of Dannenberg et al. 2015[1] posit a model by which to interpret our findings related to scopolamine–related changes in hippocampal theta oscillations observed during memory processing. Dannenberg et al. describe a model by which cholinergic neurons in the basal forebrain (BF) modulate theta oscillations in the hippocampus via a two pathway mechanism, a "direct" pathway by which cholinergic axons synapse on the hippocampus as well as an "indirect" pathway by which cholinergic BF neurons modulate neurons locally that subsequently synapse in the hippocampus. The latter may be directly linked with BF generation of theta, while the former relies on a cholinergic activation of inhibitory interneurons in the hippocampus that in turn modulate pyramidal neuron rhythmicity, favoring theta–locked firing and ongoing theta generation in the hippocampus itself.

Using single nucleus RNA-sequencing data originally published in Ayhan et al. 2021b[2], we determined the cell type specific expression of all cholinergic receptors. We find that the human hippocampus preferentially expresses CHRM3, the gene encoding cholinergic muscarinic subtype 3 receptors, in both inhibitory and pyramidal neurons of CA1 and CA3. We believe these findings support the existence of a direct pathway and partially support the model articulated in Dannenberg et al., although the concomitant expression on pyramidal neurons may favor complementary processes that underlie the impact of scopolamine on memory encoding. These may include an impact on CA1-CA3 synaptic LTP, as summarized in Ballinger et al. 2016[3]. Although RNA expression is not necessarily correlated with protein expression, the finding that human hippocampal inhibitory and pyramidal neurons exhibit greater expression of CHRM3 above all other cholinergic receptor genes may indicate a specific molecular pathway by which cholinergic modulation directly affects hippocampal function.

We acknowledge that these findings do not rule out the impact of an indirect septal–hippocampal pathway in humans, and further analysis of specific circuit dynamics (likely using in vitro human hippocampal preparations) will be needed to establish more precisely the cell and receptor populations that mediate cholinergic modulation of hippocampal rhythmicity in humans.

Supplementary Tables and Figures

Subject ID	Gender	AH	PH	MH	EC
S1	M	5	3	0	2
S2	M	4	3	0	0
S3	M	2	0	0	3
S4	M	4	4	0	4
S5	F	5	0	0	1
S6	F	2	1	3	2
S7	M	5	5	5	0
S8	F	4	3	0	4
S9	F	4	2	0	0
S10	F	3	3	0	0
S11	M	1	2	0	0
S12	M	5	3	0	0

Table S1. Subject demographics and electrode coverage. Number of electrodes across all regions of interest. AH: Anterior Hippocampus, PH: Posterior Hippocampus, MH: Mid-Hippocampus, EC: Entorhinal Cortex.

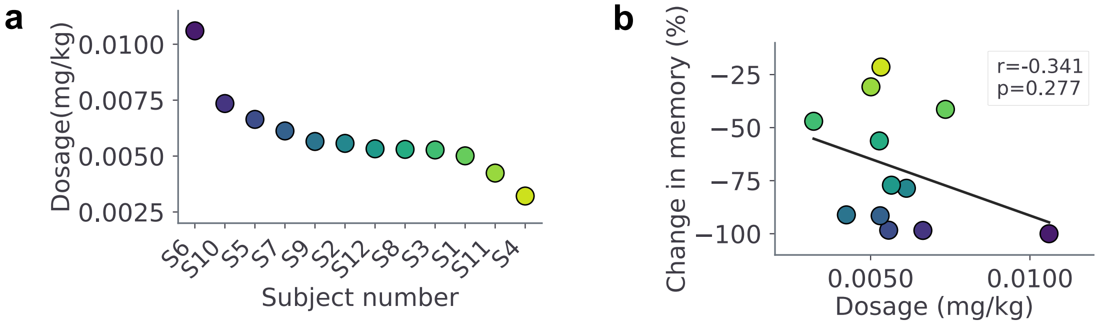

Figure S1. Effect of scopolamine concentration on memory. a. Scopolamine concentration (i.e., scopolamine dosage divided by subject's weight) for each subject. **b.** Subject-level correlation between scopolamine concentration and change in memory performance. Change in memory performance is not significantly correlated with scopolamine concentration ($r = -0.341$, $p = 0.277$, two-sided Pearson's correlation), although there was a trend for subjects with higher concentrations to have higher degrees of memory impairment.

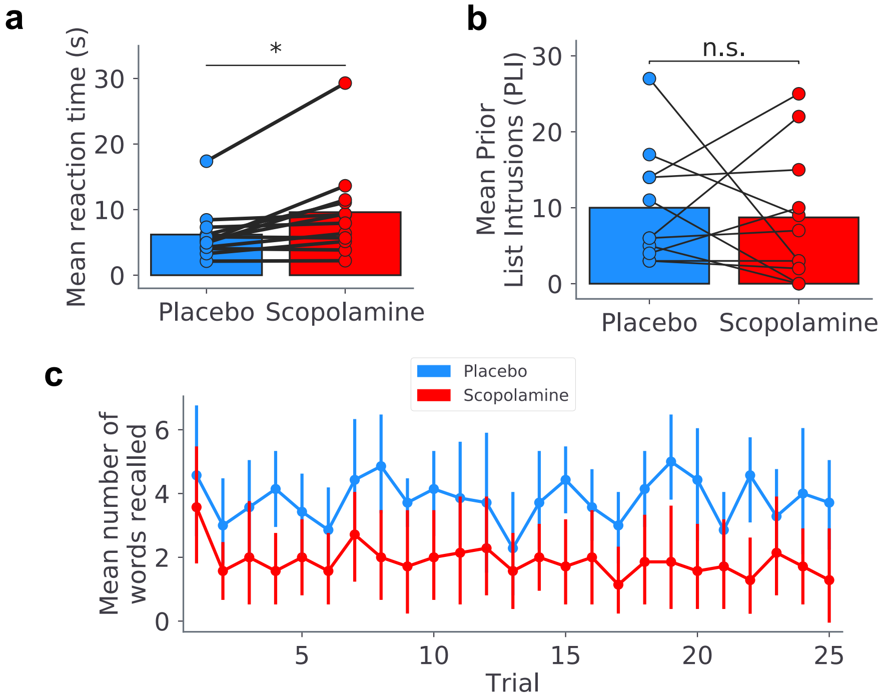

Figure S2. Additional analyses on the behavioral effects of scopolamine. a. Bar plot showing subject-level mean reaction time during the math distractor for each condition ($t(12) = -3.04$, $p = 0.01$, paired t-test). **b.** Bar plot showing subject-level mean number of prior list intrusions (PLI) for each condition ($p > 0.05$, paired t-test). **c.** Mean number of words recalled across trials. Each session was made up of 25 trials of 12 words each. Data are presented as mean values across subjects, and error bars indicate the 95% confidence interval. Source data are provided as a source data file.

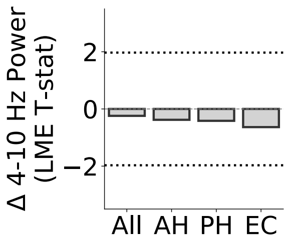

Figure S3. **Changes in fast theta power between placebo and scopolamine.** Plot showing the t-statistics of a linear mixed effects (LME) model comparing power changes between placebo and scopolamine for electrodes across all hippocampal formation subregions combined (All), anterior hippocampus (AH) only, posterior hippocampus (PH) only, and entorhinal cortex (EC) only. Dashed lines indicate 95% confidence interval. Source data are provided as a source data file.

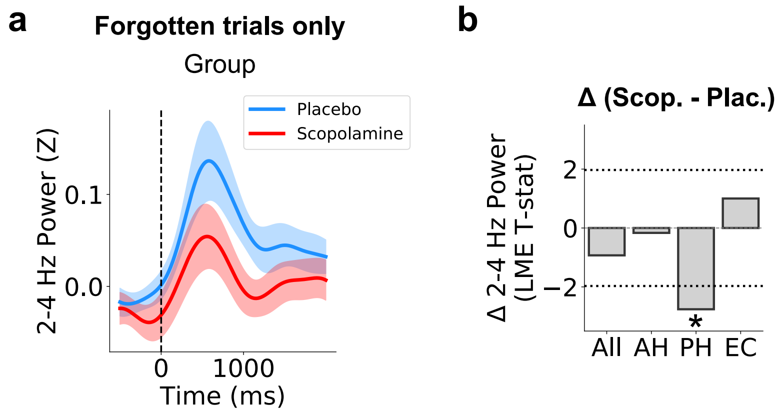

Figure S4. **Memory-evoked power effects on forgotten trials only. a.** Group level mean normalized power for the placebo (blue) and scopolamine (red) conditions when including forgotten trials only. Shading denotes ± SEM. **b.** Plot showing statistical differences in time-averaged 2–4 Hz normalized power across forgotten trials only between placebo and scopolamine for electrodes within all hippocampal formation subregions combined (All), anterior hippocampus (AH) only, posterior hippocampus (PH) only, and entorhinal cortex (EC) only. Dashed lines indicate 95% confidence intervals. Asterisk denotes statistical significance. The effect was strongest in the PH ($t(23) = -2.572$, $p = 0.010$, LME model, multiple-comparison corrected). Source data are provided as a source data file.

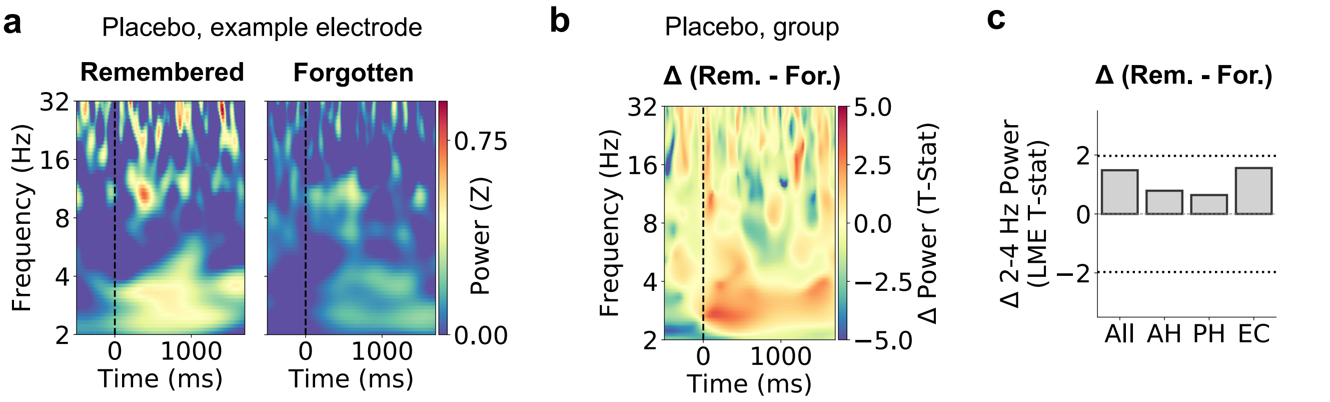

Figure S5. **Subsequent memory effect (SME) of normalized power during placebo. a.** Time–frequency spectrograms showing memory-evoked normalized power for a hippocampal electrode across all remembered (left) and forgotten (right) trials. The increase in 2–4 Hz slow theta power seen during remembered trials is not present for forgotten trials. **b.** Group-level time–frequency spectrogram showing differences in normalized power between remembered and forgotten trials. **c.** Plot showing statistical differences in time-averaged 2–4 Hz normalized power between remembered and forgotten trials for electrodes within all hippocampal formation subregions combined (All), anterior hippocampus (AH) only, posterior hippocampus (PH) only, and entorhinal cortex (EC) only. Dashed lines indicate 95% confidence intervals. Source data are provided as a source data file.

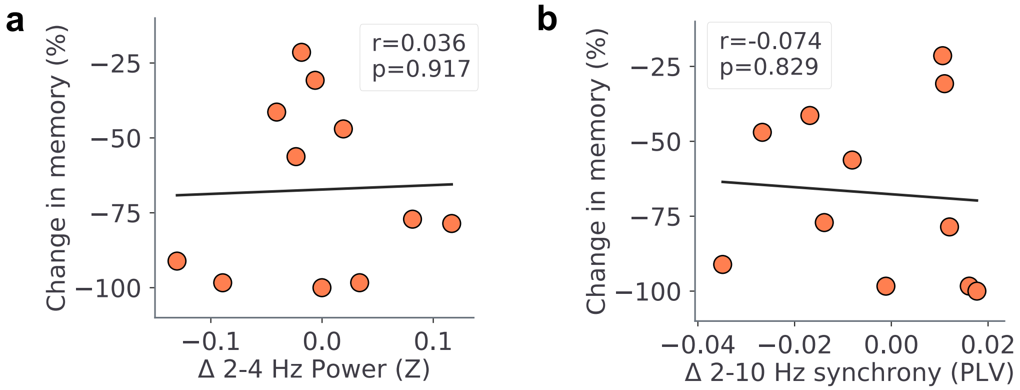

Figure S6. Effects of memory on power and synchrony. a. Subject-level correlation between changes in 2–4 Hz normalized power and changes in memory ($r = 0.036$, $p = 0.917$, two-sided Pearson's correlation). Changes in power measures do not explain changes in memory following scopolamine. **b.** Subject-level correlation between changes in 2–10 Hz synchrony measures and changes in memory ($r = -0.074$, $p = 0.829$, two-sided Pearson's correlation). Changes in synchrony measures do not explain changes in memory following scopolamine. Source data are provided as a source data file.

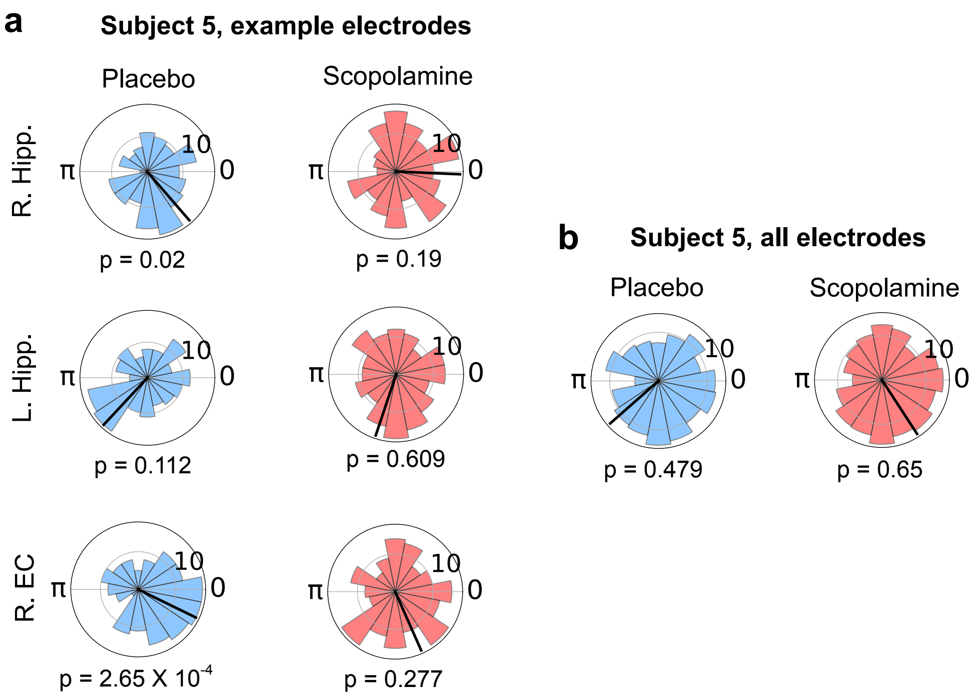

Figure S7. Individual electrodes reset to different preferred phases. a. Circular histograms of three example electrodes from subject 5, displaying phase distribution of a 8-Hz oscillation at 100 ms following encoding cue during placebo and scopolamine conditions (Rayleigh tests). Each electrode resets to a different preferred phase angle. **b.** Circular histograms showing the phase distribution across all electrodes for subject 5. Because individual electrodes reset to different preferred phase angles, the phase distribution at the subject-level is nearly uniform. Source data are provided as a source data file.

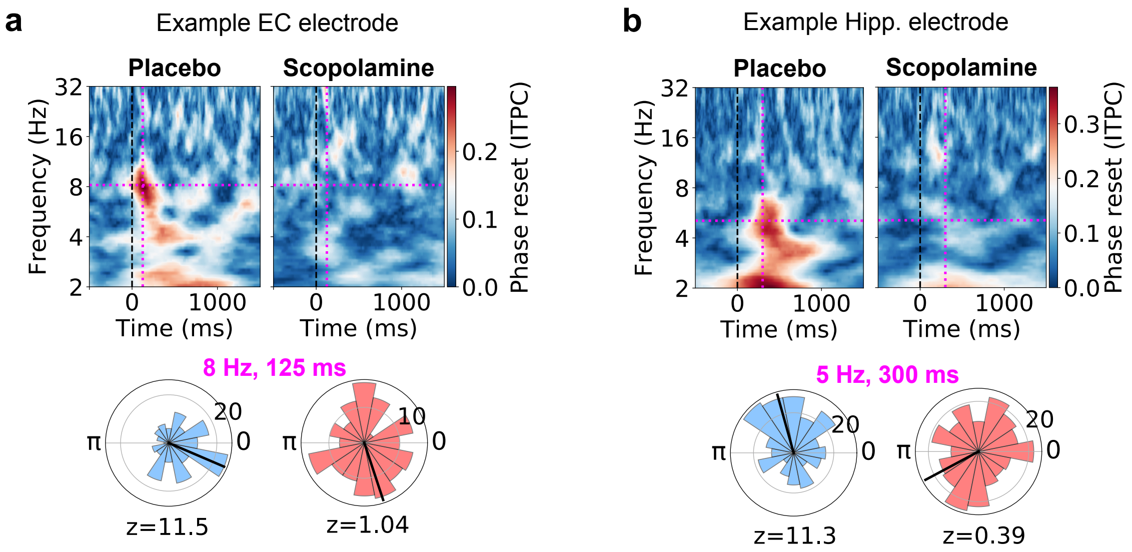

Figure S8. ITPC effects in individual electrode examples. a. Top: ITPC during encoding for an example entorhinal cortex (EC) electrode for the placebo and scopolamine conditions. The increase in 8 Hz theta phase reset seen during the placebo session is not present during the scopolamine session. Magenta dashed lines indicate peak in ITPC between 0–300ms for the placebo condition. Bottom: circular histograms showing phase distributions at the peak in ITPC following encoding cue for placebo (blue) and scopolamine (red). **b.** Top: ITPC during encoding for an example hippocampal electrode for the placebo and scopolamine conditions. The increase in 5 Hz theta phase reset seen during the placebo session is not present during the scopolamine session. Magenta dashed lines indicate peak in ITPC between 0–300ms for the placebo condition. Bottom: circular histograms showing phase distributions at the peak in ITPC following encoding cue for placebo (blue) and scopolamine (red). Source data are provided as a source data file.

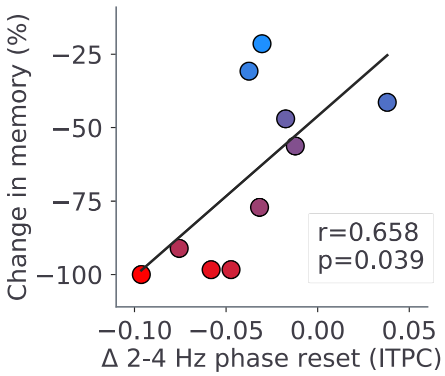

Figure S9. Relation between slow theta phase reset and severity of memory impairment from scopolamine. Correlation between changes in slow theta (2–4 Hz) phase reset and changes in memory ($r = 0.658$, $p = 0.039$, two-sided Pearson's correlation). Source data are provided as a source data file.

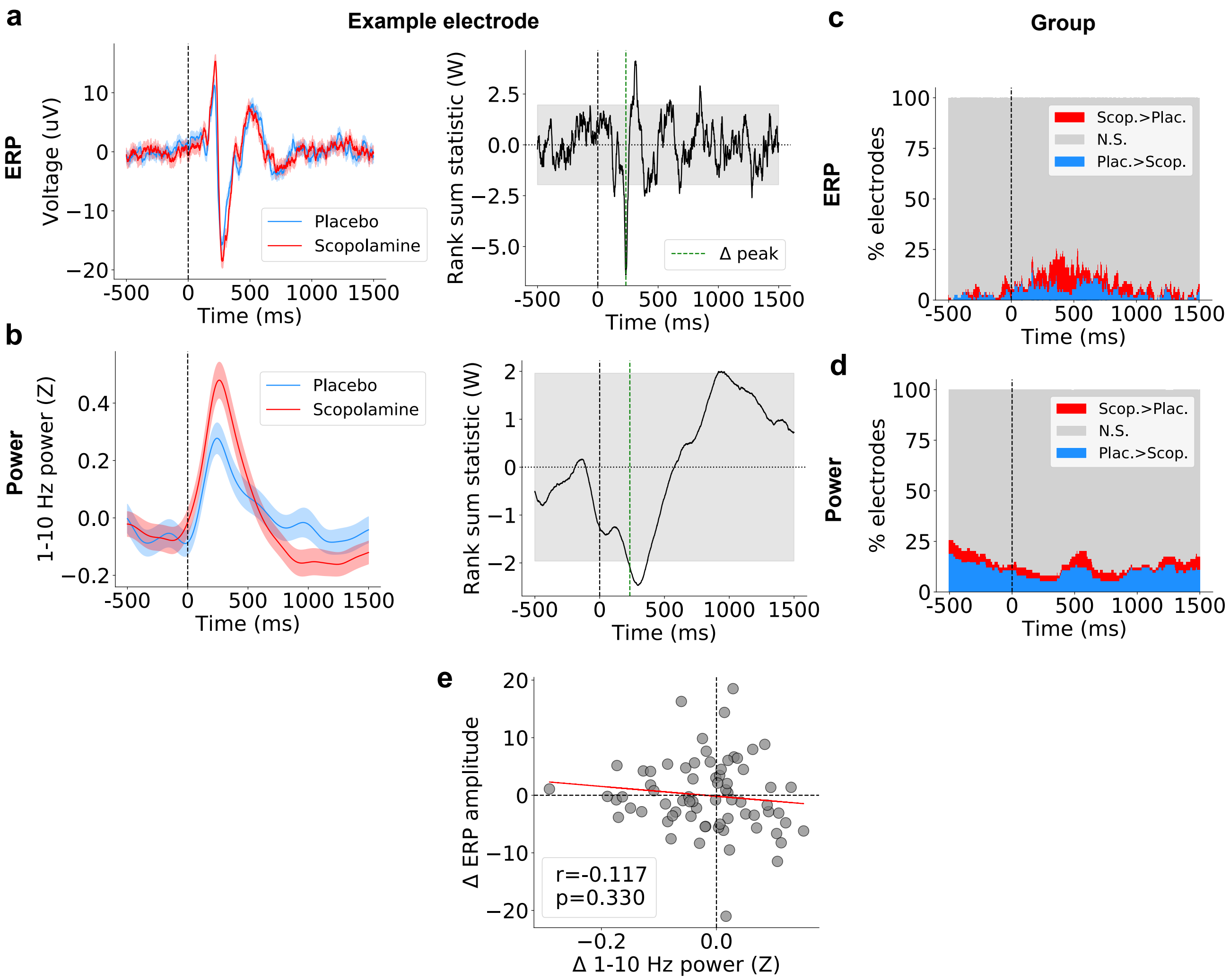

Figure S10. **Event-related potentials (ERPs) do not explain changes in power. a.** Left: mean ERP for an example EC electrode. Data are presented as mean values across trials, and shading denotes ± SEM. Right: corresponding mean ERP differences between placebo and scopolamine (Wilcoxon rank-sum test). Gray shading denotes areas of non-significant effects, and dashed green line denotes timepoint of maximal ERP difference between conditions. **b.** Left: mean 1–10-Hz power for the same example electrode shown above. Right: corresponding mean power differences between placebo and scopolamine (Wilcoxon rank-sum test). Gray shading denotes areas of non-significant effects, and dashed green line denotes timepoint of maximal ERP difference between conditions. **c.** Group-level percentage of electrodes showing significant increase for placebo (blue = Plac. > Scop.), scopolamine (red = Scop. > Plac.), or no significant changes (gray = N.S.) for ERP measures. **d.** Group-level percentage of electrodes showing significant increase for placebo (blue = Plac. > Scop.), scopolamine (red = Scop. > Plac.), or no significant changes (gray = N.S.) for power measures. The distribution of significant differences between placebo and scopolamine differs between ERP and power measures. **e.** Correlation between changes in ERP amplitude (measured at the point of maximal ERP difference between conditions, with a 150-ms buffer on each side) and changes in 1–10-Hz power at those same points in time. Peak amplitude changes in ERP do not significantly correlate with changes in power ($r = -0.117$, $p = 0.330$, two-sided Pearson's correlation). Source data are provided as a source data file.

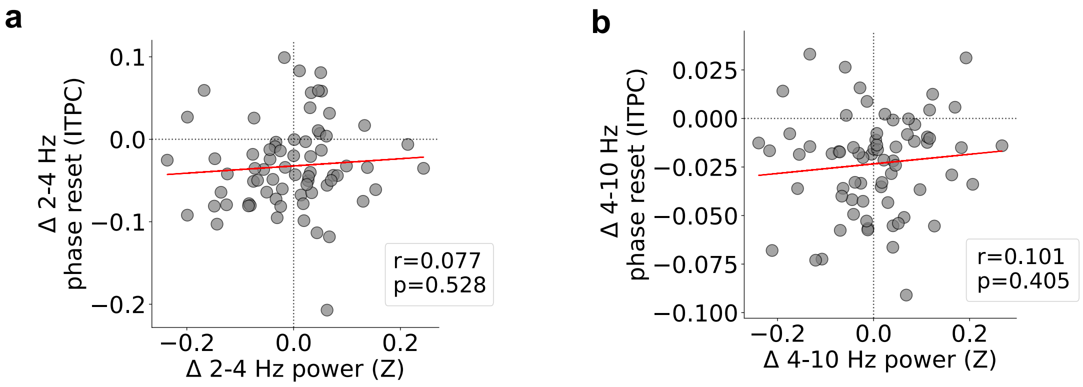

Figure S11. Changes in power do not explain changes in phase reset. a. Correlation between changes in slow theta (2–4 Hz) power and changes in slow theta phase reset across all electrodes ($r = 0.077$, $p = 0.528$, two-sided Pearson's correlation). **b.** Correlation between changes in fast theta (4–10 Hz) power and changes in fast theta phase reset across all electrodes ($r = 0.101$, $p = 0.405$, two-sided Pearson's correlation). Source data are provided as a source data file.

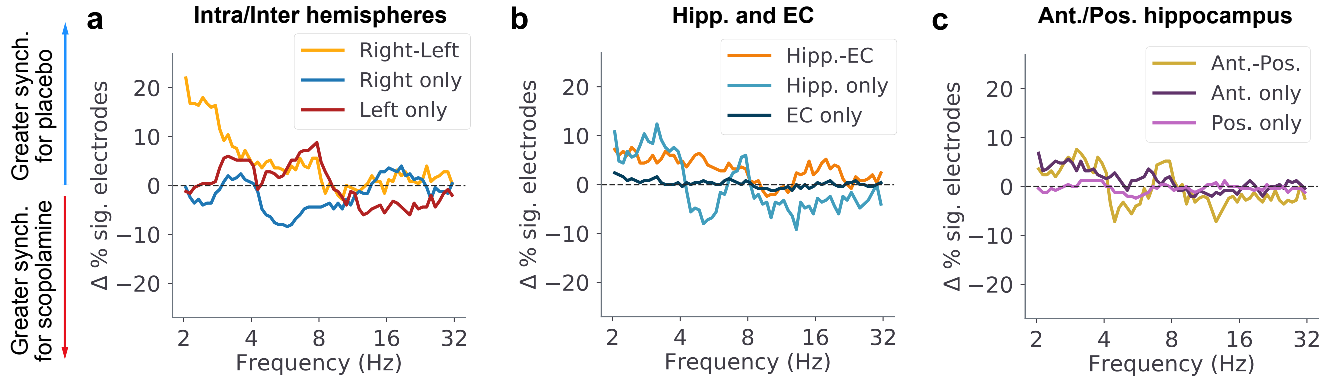

Figure S12. Connectivity disruptions within and across hemispheres and different subregions. a. Change in percentage of electrode pairs showing significantly greater connectivity for placebo and scopolamine, separated by inter-hemispheric (Right-Left) and intra-hemispheric (Right only and Left only) electrode pairs. **b.** Change in percentage of electrode pairs showing significantly greater connectivity for placebo and scopolamine, separated by hippocampus-entorhinal cortex (Hipp.-EC), hippocampus only (Hipp. only), and entorhinal cortex only (EC only) electrode pairs. **C.** Change in percentage of electrode pairs showing significantly greater connectivity for placebo and scopolamine, separated by anterior-posterior hippocampus (Ant.-Pos.), anterior hippocampus only (Ant. only), and posterior hippocampus only (Pos. only) electrode pairs. Source data are provided as a source data file.

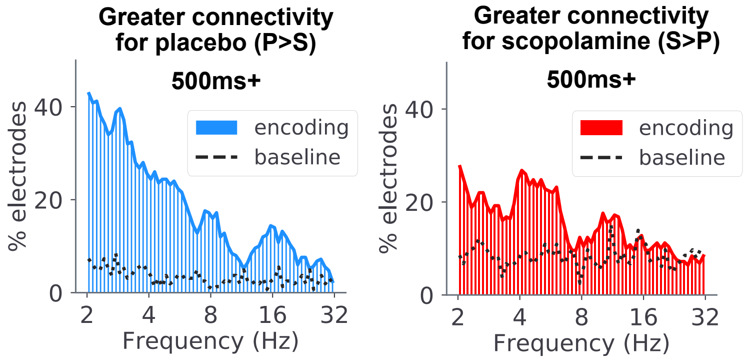

Figure S13. Changes in phase reset do not explain changes in synchrony. Percentage of significant electrodes showing increase connectivity for placebo (left; blue) and scopolamine (right; red) after excluding the first 500 ms following word onset where the phase reset effect is strongest ($z = -3.94$, $p < 10^{-4}$, two-proportion z-test). Dotted lines indicate the percentage of electrodes showing significant increases in synchrony during baseline. Source data are provided as a source data file.

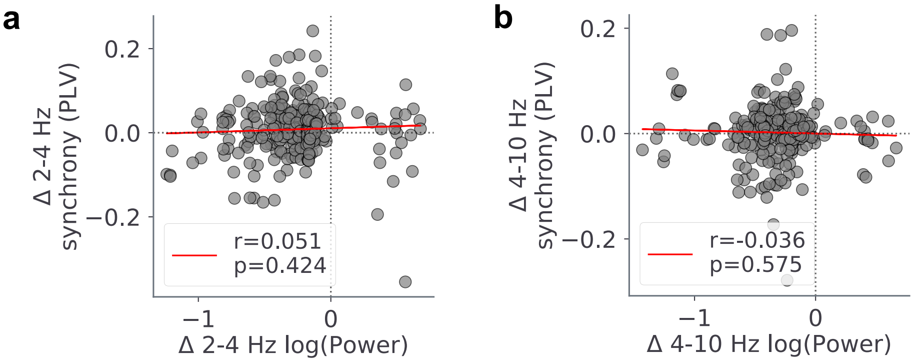

Figure S14. Changes in power do not explain changes in synchrony. a. Correlation between changes in slow theta (2–4 Hz) power and changes in slow theta synchrony across all electrode pairs ($r = 0.051$, $p = 0.424$, two-sided Pearson's correlation). **b.** Correlation between changes in fast theta (4–10 Hz) power and changes in fast theta synchrony across all electrode pairs ($r = -0.036$, $p = 0.575$, two-sided Pearson's correlation). Source data are provided as a source data file.

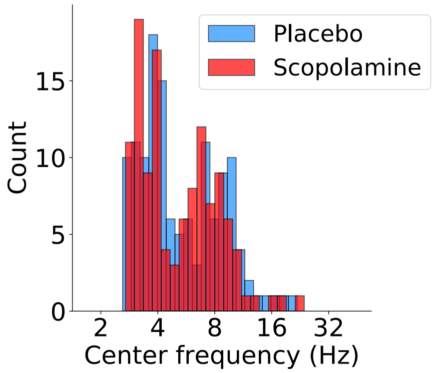

Figure S15. Distribution of peak frequencies does not differ between conditions. Distribution of non-baseline-corrected power peak frequencies for placebo and scopolamine ($p > 0.05$, Wilcoxon rank-sum test). Source data are provided as a source data file.

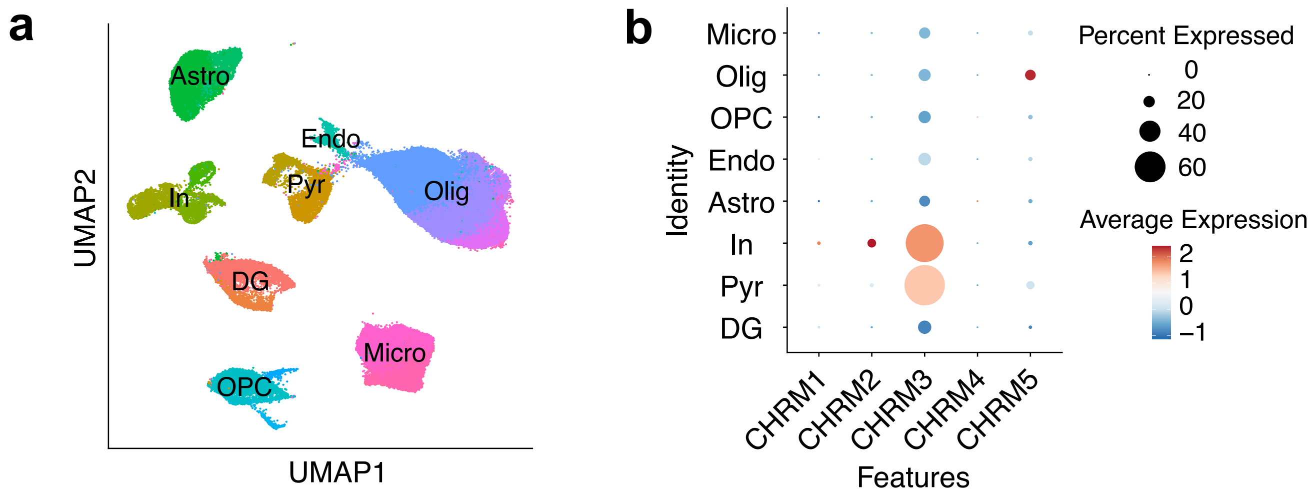

Figure S16. Gene expression in the human hippocampus. 125,583 cells from anterior and posterior hippocampal tissue samples from five patients[2]. **a.** UMAP plot of hippocampal cells colored by Seurat cluster and cell-type annotation. DG = dentate gyrus neurons, Pyr = pyramidal cells, In = inhibitory neurons, Astro = astrocytes, Endo = endothelial cells, OPC = oligodendrocyte precursor cells, Olig = oligodendrocytes, Micro = microglia. **b.** Muscarinic acetylcholine receptor gene expression in major hippocampal cell clusters. Of the genes encoding the five muscarinic acetylcholine receptor subtypes, CHRM3 (the gene encoding the muscarinic M3 receptor subtype) is preferentially expressed in the hippocampus. CHRM3 is primarily expressed in inhibitory and pyramidal cell clusters.

Supplementary References

1. Dannenberg, H. *et al.* Synergy of direct and indirect cholinergic septo-hippocampal pathways coordinates firing in hippocampal networks. *J. Neurosci.* **35**, 8394–8410 (2015).

2. Ayhan, F. *et al.* Resolving cellular and molecular diversity along the hippocampal anterior-to-posterior axis in humans. *Neuron* **109**, 2091–2105.e6 (2021).

3. Ballinger, E. C., Ananth, M., Talmage, D. A. & Role, L. W. Basal Forebrain Cholinergic Circuits and Signaling in Cognition and Cognitive Decline. *Neuron* **91**, 1199–1218 (2016).
