# Supplementary material for: Acetylcholine modulates the temporal dynamics of human theta oscillations during memory
Source: Nat Commun. 2023 Aug 30;14:5283. doi: 10.1038/s41467-023-41025-y (PMC10469188; doi:10.1038/s41467-023-41025-y)
Supplement: Supplementary file 3 — Reporting Summary [file 41467_2023_41025_MOESM3_ESM.pdf]

Corresponding author(s): Joshua Jacobs
Last updated by author(s): Jul 21, 2023

# Reporting Summary

Nature Portfolio wishes to improve the reproducibility of the work that we publish. This form provides structure for consistency and transparency in reporting. For further information on Nature Portfolio policies, see our Editorial Policies and the Editorial Policy Checklist.

## Statistics

For all statistical analyses, confirm that the following items are present in the figure legend, table legend, main text, or Methods section.

| n/a | Confirmed | |
|---|---|---|
| ☐ | ☒ | The exact sample size (*n*) for each experimental group/condition, given as a discrete number and unit of measurement |
| ☐ | ☒ | A statement on whether measurements were taken from distinct samples or whether the same sample was measured repeatedly |
| ☐ | ☒ | The statistical test(s) used AND whether they are one- or two-sided<br>*Only common tests should be described solely by name; describe more complex techniques in the Methods section.* |
| ☐ | ☒ | A description of all covariates tested |
| ☐ | ☒ | A description of any assumptions or corrections, such as tests of normality and adjustment for multiple comparisons |
| ☐ | ☒ | A full description of the statistical parameters including central tendency (e.g. means) or other basic estimates (e.g. regression coefficient) AND variation (e.g. standard deviation) or associated estimates of uncertainty (e.g. confidence intervals) |
| ☐ | ☒ | For null hypothesis testing, the test statistic (e.g. *F*, *t*, *r*) with confidence intervals, effect sizes, degrees of freedom and *P* value noted<br>*Give P values as exact values whenever suitable.* |
| ☒ | ☐ | For Bayesian analysis, information on the choice of priors and Markov chain Monte Carlo settings |
| ☒ | ☐ | For hierarchical and complex designs, identification of the appropriate level for tests and full reporting of outcomes |
| ☐ | ☒ | Estimates of effect sizes (e.g. Cohen's *d*, Pearson's *r*), indicating how they were calculated |

*Our web collection on statistics for biologists contains articles on many of the points above.*

## Software and code

Policy information about availability of computer code

| | |
|---|---|
| Data collection | The task was coded using the publicly available Python Experiment-Programming Library (PyEPL; https://pyepl.sourceforge.net/), which allows for presentation of visual stimuli and supports both manual (keyboard) and sound (microphone) input as responses. For ensuring precise timing, logging, and communication with external hardware, we used the publicly available UnityEPL (https://github.com/pennmem/UnityEPL). |
| Data analysis | All data analyses were performed in MATLAB (R2018a) or Python (3.6), using publicly available software for behavioral analysis of free recall data (https://github.com/pennmem/pybeh) and human electrophysiology data (https://github.com/pennmem/ptsa and https://github.com/pennmem/cmlreaders). Other custom code is available at https://github.com/tgedankien/scopolamine. Gene sequences were aligned and mapped using CellRanger software (v.3.0.2, 10X Genomics). |

For manuscripts utilizing custom algorithms or software that are central to the research but not yet described in published literature, software must be made available to editors and reviewers. We strongly encourage code deposition in a community repository (e.g. GitHub). See the Nature Portfolio guidelines for submitting code & software for further information.

## Data

Policy information about availability of data

All manuscripts must include a data availability statement. This statement should provide the following information, where applicable:

- Accession codes, unique identifiers, or web links for publicly available datasets
- A description of any restrictions on data availability
- For clinical datasets or third party data, please ensure that the statement adheres to our policy

The data that support the findings of this study are provided in the Source Data file.

## Human research participants

Policy information about studies involving human research participants and Sex and Gender in Research.

| | |
|---|---|
| Reporting on sex and gender | The study included 7 males and 5 females. Sex was determined based on participant's medical chart. No sex- or gender-based analysis were performed in the study as they were not part of the a priori hypotheses being tested. |
| Population characteristics | Covariate-relevant population characteristics included age, seizure onset zones and weight. Genotypic information was not relevant for our analysis. All subjects were surgical epilepsy patients. |
| Recruitment | Participants were adult patients undergoing invasive electroencephalographic (iEEG) monitoring as part of a standard clinical procedure for the treatment of pharmacologically resistant epilepsy at the University of Texas Southwestern (UTSW). Potential participants were identified from the pool of patients presented at the epilepsy management conference, and approached when they presented to the clinic for evaluation for seizure surgery. Of the larger population of subjects available for this study, participants were those who were capable of participating in episodic memory paradigms and agreed to participate in memory related research. |
| Ethics oversight | The protocol was approved by the UTSW Institutional Review Board on Human Subjects Research prior to data collection. All participants provided informed written consent. |

Note that full information on the approval of the study protocol must also be provided in the manuscript.

# Field-specific reporting

Please select the one below that is the best fit for your research. If you are not sure, read the appropriate sections before making your selection.

☒ Life sciences ☐ Behavioural & social sciences ☐ Ecological, evolutionary & environmental sciences

For a reference copy of the document with all sections, see nature.com/documents/nr-reporting-summary-flat.pdf

# Life sciences study design

All studies must disclose on these points even when the disclosure is negative.

| | |
|---|---|
| Sample size | This study involved within-subject analyses. Sample size was determined by the number of memory encoding events per session (ranging from 60 to 300) and the number of electrodes within each region being studied (ranging from 3 to 15). The sample sizes used in our study are similar to sample sizes used in other published work examining theta oscillations in the MTL during episodic memory (Sederberg et al., 2003; Lega et al., 2012). |
| Data exclusions | Electrodes displaying radiographic abnormalities including temporal sclerosis or previous neurosurgery were excluded from analysis. A kurtosis algorithm was used to exclude abnormal events and interictal activity. One subject was excluded from all neural analyses due to excessive noise. One subject was excluded from the phase reset analysis due to a low number of trials in the drug condition. |
| Replication | This study was not yet replicated because only a limited number of participants was recruited due to clinical limitations. |
| Randomization | The order of drug/placebo sessions was randomized within subjects. |
| Blinding | The test administrator and participant were blinded to the drug/placebo randomization. |

# Reporting for specific materials, systems and methods

We require information from authors about some types of materials, experimental systems and methods used in many studies. Here, indicate whether each material, system or method listed is relevant to your study. If you are not sure if a list item applies to your research, read the appropriate section before selecting a response.

## Materials & experimental systems

| n/a | Involved in the study |
|---|---|
| ☒ | ☐ Antibodies |
| ☒ | ☐ Eukaryotic cell lines |
| ☒ | ☐ Palaeontology and archaeology |
| ☒ | ☐ Animals and other organisms |
| ☒ | ☐ Clinical data |
| ☒ | ☐ Dual use research of concern |

## Methods

| n/a | Involved in the study |
|---|---|
| ☒ | ☐ ChIP-seq |
| ☒ | ☐ Flow cytometry |
| ☒ | ☐ MRI-based neuroimaging |
